# Supplementary material for: Rhotekin regulates axon regeneration through the talin–Vinculin–Vinexin axis in Caenorhabditis elegans
Source: PLoS Genet. 2023 Dec 27;19(12):e1011089. doi: 10.1371/journal.pgen.1011089 (PMC10752531; doi:10.1371/journal.pgen.1011089)
Supplement: S1 Fig — The nucleotides and corresponding amino acids around the deleted regions are shown. The rtkn-1(km94) mutation is a 2-bp deletion that causes a frameshift (amino acids in red) and a premature stop codon (*). (PDF) [file pgen.1011089.s001.pdf]

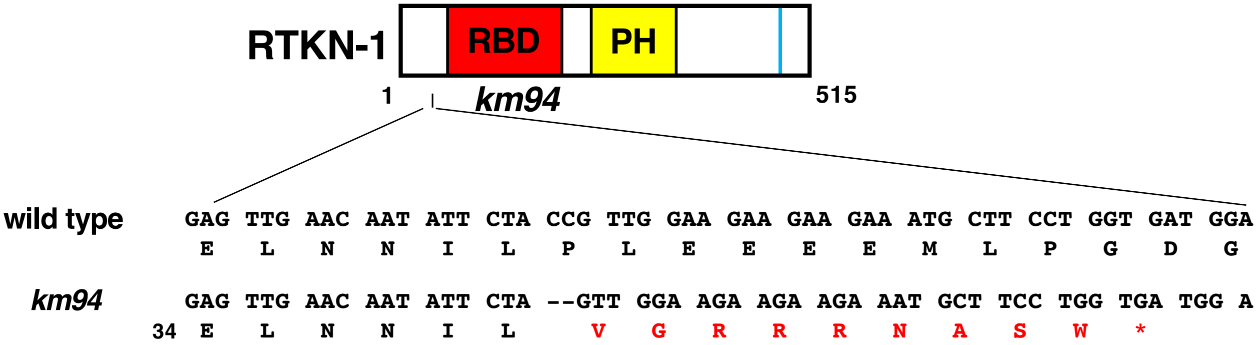
RTKN-1
RBD
PH
1
km94
515
wild type
GAG TTG AAC AAT ATT CTA CCG TTG GAA GAA GAA GAA ATG CTT CCT GGT GAT GGA
E L N N I L P L E E E E M L P G D G
km94
GAG TTG AAC AAT ATT CTA --GTT GGA AGA AGA AGA AAT GCT TCC TGG TGA TGG A
34 E L N N I L V G R R R N A S W *
