# Supplementary material for: Rhotekin regulates axon regeneration through the talin–Vinculin–Vinexin axis in Caenorhabditis elegans
Source: PLoS Genet. 2023 Dec 27;19(12):e1011089. doi: 10.1371/journal.pgen.1011089 (PMC10752531; doi:10.1371/journal.pgen.1011089)
Supplement: S2 Fig — The nucleotides and corresponding amino acids around the deleted region are shown. The inserted nucleotides are shown in orange. The sorb-1(gk304) mutation is a 430-bp deletion and a 2-bp insertion, resulting in a frameshift (amino acids in red) and a premature stop codon (*). (PDF) [file pgen.1011089.s002.pdf]

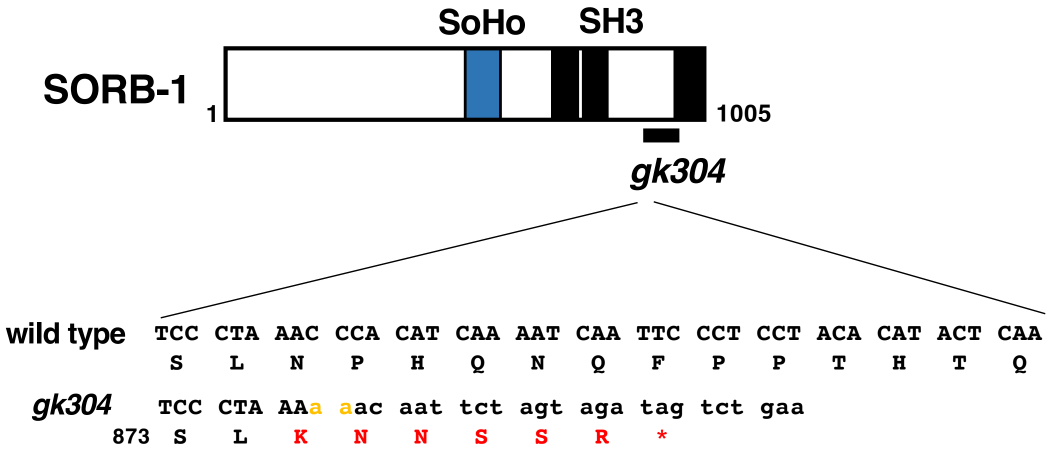
SoHo
SH3
SORB-1
1
1005
gk304
wild type
TCC CTA AAC CCA CAT CAA AAT CAA TTC CCT CCT ACA CAT ACT CAA
S L N P H Q N Q F P P T H T Q
gk304
TCC CTA AAa aac aat tct agt aga tag tct gaa
873
S L K N N S S R *
