# Supplementary figures and images for: Rhotekin regulates axon regeneration through the talin–Vinculin–Vinexin axis in Caenorhabditis elegans
Source: PLoS Genet. 2023 Dec 27;19(12):e1011089. doi: 10.1371/journal.pgen.1011089 (PMC10752531; doi:10.1371/journal.pgen.1011089)

**human vinculin**

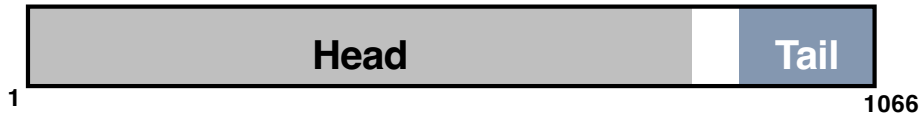

**DEB-1**

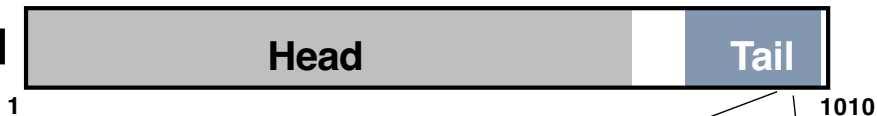

vinculin 972 **CTDKRIRTNLLQV** 984  
DEB-1 906 **CTDIKMRTALLQV** 918

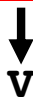

*deb-1(gk329549)*

Supplement: S3 Fig — The domain structure of DEB-1 and human vinculin is shown. The deb-1(gk329549) allele harbors the D908V mutation in the tail domain. Identical and similar residues are highlighted in red and yellow shading, respectively. (PDF) [file pgen.1011089.s003.pdf]
