# Supplementary material for: Rhotekin regulates axon regeneration through the talin–Vinculin–Vinexin axis in Caenorhabditis elegans
Source: PLoS Genet. 2023 Dec 27;19(12):e1011089. doi: 10.1371/journal.pgen.1011089 (PMC10752531; doi:10.1371/journal.pgen.1011089)
Supplement: S4 Fig — The genomic structure of the alp-1 gene is shown. The nucleotides and corresponding amino acids around the deleted regions are also shown. Nucleotides derived from exonic and intronic regions are indicated by upper- and lower-case letters, respectively. The inserted nucleotides are shown in orange. The alp-1(ok820) mutation is a 1,236-bp deletion, resulting in a splicing change that skips an exon and produces a short ALP-1 protein missing 57 amino acids. The alp-1(km95) mutation is a 21-bp deletion and a 3-bp insertion, resulting in a frameshift (amino acid in red) and a premature stop codon (*). (PDF) [file pgen.1011089.s004.pdf]

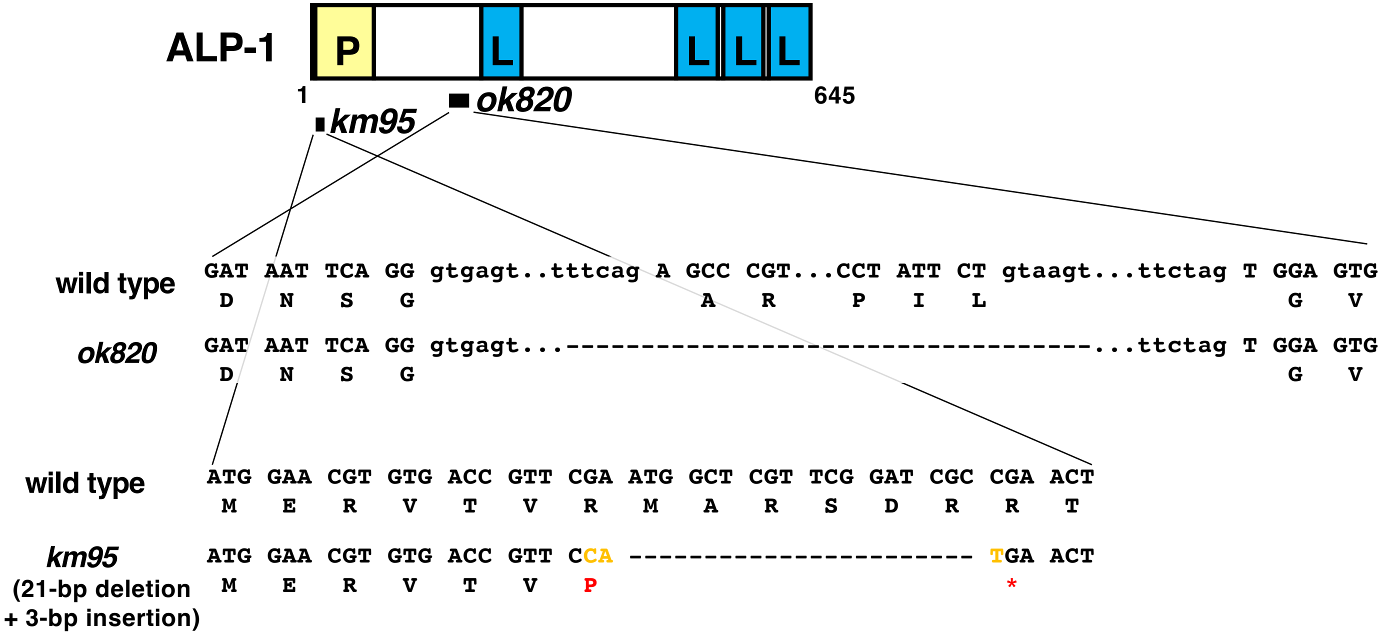

ALP-1
P
L
L
L
L
1
645
ok820
km95
wild type
GAT AAT TCA GG gtgagt..tttcag A GCC CGT...CCT ATT CT gtaagt...ttctag T GGA GTG
D N S G A R P I L G V
ok820
GAT AAT TCA GG gtgagt...-----------------------------------...ttctag T GGA GTG
D N S G G V
wild type
ATG GAA CGT GTG ACC GTT CGA ATG GCT CGT TCG GAT CGC CGA ACT
M E R V T V R M A R S D R R T
km95
(21-bp deletion
+ 3-bp insertion)
ATG GAA CGT GTG ACC GTT CCA ------------------------ TGA ACT
M E R V T V P *
