# Supplementary material for: Rhotekin regulates axon regeneration through the talin–Vinculin–Vinexin axis in Caenorhabditis elegans
Source: PLoS Genet. 2023 Dec 27;19(12):e1011089. doi: 10.1371/journal.pgen.1011089 (PMC10752531; doi:10.1371/journal.pgen.1011089)
Supplement: S1 Table — (PDF) [file pgen.1011089.s005.pdf]

S1 Table. Raw data for genotypes tested by axotomy.

Strain	Genotype (*juIs76* background)	No. of axons	No. of re-generations (% of total)	*p*-value (Bonferroni-adjusted)	Compared with
KU501[a]	wild type	51	35 (69%)	-	-
KU1636[a]	*rtkn-1(ok1404)*	51	20 (39%)	0.0104	KU501[a]
KU1637[a]	*rtkn-1(km94)*	56	23 (41%)	0.0126	KU501[a]
KU1638	*rtkn-1(ok1404); kmEx1638 [Prtkn-1::rtkn-1]*	51	37 (73%)	0.0024	KU1636[a]
KU1639	*rtkn-1(ok1404); kmEx1639 [Punc-25::rtkn-1]*	54	34 (63%)	0.0386	KU1636[a]
KU1640	*rtkn-1(ok1404); kmEx1640 [Pmec-7::rtkn-1]*	50	18 (36%)	0.8379	KU1636[a]
KU1641	*rtkn-1(ok1404); kmEx1641 [Punc-25::rho-1(G14V)]*	53	22 (42%)	0.8437	KU1636[a]
KU1642	*rtkn-1(km94); kmEx1406 [Punc-25::venus::mlc-4(DD)]*	45	22 (49%)	0.5460	KU1637[a]
KU501[b]	wild type	55	40 (73%)	-	-
KU1632[a]	*sorb-1(gk304)*	40	17 (43%)	0.0110	KU501[b]
KU1636[b]	*rtkn-1(ok1404)*	38	14 (37%)	0.0014	KU501[b]
KU1643	*sorb-1(gk304); rtkn-1(ok1404)*	42	17 (40%)	1.0000	KU1632[a]
				0.8200	KU1636[b]
KU1635	*sorb-1(gk304); kmEx1406 [Punc-25::venus::mlc-4(DD)]*	59	27 (46%)	0.8376	KU1632[a]
KU501[c]	wild type	74	50 (68%)	-	-
KU1375	*deb-1(gk329549)*	58	21 (36%)	0.0008	KU501[c]
KU1632[b]	*sorb-1(gk304)*	61	27 (44%)	0.0174	KU501[c]
KU1633	*deb-1(gk329549) sorb-1(gk304)*	73	35 (48%)	0.7293	KU1632[b]
KU501[d]	wild type	80	51 (64%)	-	-
KU1411	*alp-1(ok820)*	56	23 (41%) [1]	0.0280	KU501[d]
KU1415	*alp-1(ok820); kmEx1406 [Punc-25::venus::mlc-4(DD)]*	50	31 (62%) [1]	0.0348	KU1411
KU1644	*alp-1(km95)*	49	21 (43%)	0.0282	KU501[d]
KU1645	*alp-1(km95); kmEx1406 [Punc-25::venus::mlc-4(DD)]*	53	25 (47%)	0.6944	KU1644
KU1637[b]	*rtkn-1(km94)*	56	23 (41%)	-	-
KU1646	*alp-1(km95); rtkn-1(km94)*	54	19 (35%)	0.5607	KU1637[b]
KU501[e]	wild type	49	33 (67%)	-	-
KU1358	*tln-1(e259)*	47	17 (36%)	0.0080	KU501[e]
KU1630	*tln-1(A2534T)*	48	18 (38%)	0.0088	KU501[e]
KU1631	*tln-1(e259; T2534A)*	65	38 (58%)	0.0364	KU1630

a to e: different controls of the same strain.

Reference

1. Shimizu T, Pastuhov SI, Hanafusa H, Matsumoto K, Hisamoto N. The C. elegans BRCA2-ALP/Enigma complex regulates axon regeneration via a Rho GTPase-ROCK-MLC phosphorylation pathway. Cell Rep. 2018; 24: 1880-1889.
