# Supplementary material for: Rhotekin regulates axon regeneration through the talin–Vinculin–Vinexin axis in Caenorhabditis elegans
Source: PLoS Genet. 2023 Dec 27;19(12):e1011089. doi: 10.1371/journal.pgen.1011089 (PMC10752531; doi:10.1371/journal.pgen.1011089)
Supplement: S2 Table — (PDF) [file pgen.1011089.s006.pdf]

S2 Table. Strains used in this study.

Strain	Genotype
KU501	*juIs76 II*
KU1358	*tln-1(e259) I; juIs76 II*
KU1375	*juIs76 II; deb-1(gk329549) IV*
KU1411	*juIs76 II; alp-1(ok820) IV*
KU1415	*juIs76 II; alp-1(ok820) IV; kmEx1406 [Punc-25::venus::mlc-4(DD)]*
KU1630	*tln-1(A2534T) I; juIs76 II*
KU1631	*tln-1(e259; T2534A) I; juIs76 II*
KU1632	*juIs76 II; sorb-1(gk304) IV*
KU1633	*juIs76 II; deb-1(gk329549) sorb-1(gk304) IV*
KU1635	*juIs76 II; sorb-1(gk304) IV; kmEx1406 [Punc-25::venus::mlc-4(DD)]*
KU1636	*juIs76 II; rtkn-1(ok1404) X*
KU1637	*juIs76 II; rtkn-1(km94) X*
KU1638	*juIs76 II; rtkn-1(ok1404) X; kmEx1638 [Prtkn-1::rtkn-1]*
KU1639	*juIs76 II; rtkn-1(ok1404) X; kmEx1639 [Punc-25::rtkn-1]*
KU1640	*juIs76 II; rtkn-1(ok1404) X; kmEx1640 [Pmec-7::rtkn-1]*
KU1641	*juIs76 II; rtkn-1(ok1404) X; kmEx1641 [Punc-25::rho-1(G14V)]*
KU1642	*juIs76 II; rtkn-1(km94) X; kmEx1406 [Punc-25::venus::mlc-4(DD)]*
KU1643	*juIs76 II; sorb-1(gk304) IV; rtkn-1(ok1404) X*
KU1644	*juIs76 II; alp-1(km95) IV*
KU1645	*juIs76 II; alp-1(km95) IV; kmEx1406 [Punc-25::venus::mlc-4(DD)]*
KU1646	*juIs76 II; alp-1(km95) IV; rtkn-1(km94) X*
