# Supplementary material for: Rhotekin regulates axon regeneration through the talin–Vinculin–Vinexin axis in Caenorhabditis elegans
Source: PLoS Genet. 2023 Dec 27;19(12):e1011089. doi: 10.1371/journal.pgen.1011089 (PMC10752531; doi:10.1371/journal.pgen.1011089)
Supplement: Attachment — Submitted filename: Response to Reviewers.docx [file pgen.1011089.s007.docx]

Dear Dr. Ashrafi and Dr. Copenhaver,

Enclosed please find our revised manuscript entitled: “Rhotekin regulates axon regeneration through the talin–vinculin–vinexin axis in *Caenorhabditis elegans*” (by Sakai et al., PGENETICS-D-23-01076). Thank you for your e-mail of November 22, 2023, and the reviewers' comments on our manuscript. We appreciate these comments as they have helped us to improve the manuscript. We have revised the manuscript to address the reviewers' comments. We hope that this revised manuscript is now acceptable for publication in *PLoS Genet.* Our point-by-point responses to the reviewers’ comments are provided below.

With best regards,

Kunihiro Matsumoto

Paper: PGENETICS-D-23-01076

Authors: Sakai et al.,

Title: Rhotekin regulates axon regeneration through the talin–vinculin–vinexin axis in *Caenorhabditis elegans*

The manuscript has been revised in accordance with the comments given by the referees. The responses to the comments are as follows:

**Our responses to the comments of Referee #1**

**1) The authors use yeast two hybrid to show that it is the SH3 domain of SORB-1 likely interacts with the PxxP motif of RTKN-1. But this direct interaction is not shown. While it is likely given what is known about these domains, the authors could suggest alternatives in the absence of showing the interaction..**

As suggested, we have revised the manuscript (p. 8–9, lines 171–174; p. 10, line 220; p. 15, lines 334–335). In brief, we have softened our assumption regarding the PxxP motif of RTKN-1 as the interaction site for the SH3 domain of SORB-1.

**2) The discussion of ALP-1 and TLN-1 (last two results sections) could be explained in a more structured manner. These are complex experiments and interpretations. It might help to break the individual into discrete sections, each with its own heading and conclusion. As presented, it is difficult to follow.**

As suggested, we have divided the ALP-1 results section into three distinct parts, each with its own heading and conclusion (p. 10–12, lines 206–266). We have also revised the TLN-1 results section, particularly the heading (p. 12–13, lines 268–281). We believe that this revision has improved the flow and clarity of our results description.
